# Atopic Dermatitis Severity, Patient Perception of the Disease, and Personality Characteristics: How Are They Related to Quality of Life?

**DOI:** 10.3390/life11121434

**Published:** 2021-12-20

**Authors:** Liborija Lugović-Mihić, Jelena Meštrović-Štefekov, Iva Ferček, Nives Pondeljak, Elvira Lazić-Mosler, Ana Gašić

**Affiliations:** 1 Department of Dermatovenereology, University Hospital Center Sestre Milosrdnice, 10000 Zagreb, Croatia; jms@globalnet.hr (J.M.-Š.); iva.fercek@gmail.com (I.F.); nives.pondeljak@gmail.com (N.P.); anagasic4@gmail.com (A.G.); 2 School of Dental Medicine, University of Zagreb, 10000 Zagreb, Croatia; 3 School of Medicine, Catholic University of Croatia, 10000 Zagreb, Croatia; elvira.lazic@gmail.com; 4 Department of Dermatology and Venereology, General Hospital Sisak, 44000 Sisak, Croatia

**Keywords:** atopic dermatitis, atopic eczema, quality of life, disease severity, illness perception, Dermatology Life Quality Index, disease control, personality, Crown–Crisp Experiential Index, anxiety

## Abstract

Introduction: Atopic dermatitis (AD) is a chronic, relapsing inflammatory skin condition that greatly affects patients’ quality of life, psychological condition, and social relationships. Materials And Methods: To analyze different aspects of AD patients’ quality of life, we used the SCORing Atopic Dermatitis (SCORAD) index (for AD severity), the Dermatology Life Quality Index (DLQI), the World Health Organization Quality of Life Brief Version (WHOQOL-BREF), the Brief Illness Perception Questionnaire (Brief IPQ), and the Crown–Crisp Experiential Index (CCEI) to analyze personality traits. The study included 84 AD patients, 42 with clinical manifestations and 42 in remission. Results: SCORAD values correlated positively and linearly with DLQI (r = 0.551; *p* < 0.001) and with disease impact on life, disease control, and disease symptoms (r = 0.350–0.398; *p* ≤ 0.023). DLQI was also related to certain personality characteristics (free-floating anxiety disorder, obsession, somatization, and depression (*p* ≤ 0.032)). Symptomatic AD patients had a significantly more impaired DLQI than asymptomatic patients (*p* < 0.001) and the two groups differed in some IPQ dimensions, but they did not differ significantly concerning the WHOQOL-BREF dimensions and personality traits (CCEI). Conclusion: Since AD patient quality of life was dependent not only on disease severity but was also influenced by patient personality characteristics (anxiety disorder, obsession, somatization, depression), many factors need to be taken into account to create effective, patient-specific therapy regimens.

## 1. Introduction

Atopic dermatitis (AD) is a chronic, relapsing inflammatory skin condition that greatly affects patients’ quality of life, psychological condition, and social relationships [1]. The disease typically manifests with eczematous lesions and itching and is characterized by several pathogenetic features, such as dysfunction of the epidermal barrier with skin impairment due to the mutation of skin proteins and immune response disbalance [2,3,4,5]. In addition, various subtypes of AD, including different clinical patterns and clinical phenotypes, especially in adult AD patients, have been recorded and described and should be taken into account [5]. Patients often single out itching as the main symptom of the disease, but other symptoms are also common (itching, 54.4%; excessive dryness and scaling, 19.6%; and inflamed skin, 7.2%) [6]. Sleep cycle disorders and stigmatization, known to accompany chronic diseases, are also common problems for those with AD. These symptoms and their accompanying challenges impair the quality of life of patients and their families in many areas, including social functioning, work productivity, emotional and mental health, and physical activity. Thus, AD often broadly limits the patient’s lifestyle (51.3%) and activities (43.3%) and can lead to avoidance of social interactions (39.1%) [6,7]. Consequently, patients often struggle with various mental disorders and illnesses, e.g., adult patients have a higher risk of developing depression. Furthermore, in children with severe AD, there is an increased incidence of depression, anxiety, behavioral disorders, and autism; in adolescents with AD, the risk of developing attention deficit hyperactivity disorder (ADHD) is 1.5 times higher on average [8,9,10].

Measuring this impact on patients and their quality of life is necessary so that clinicians can manage the disease effectively with appropriately tailored plans for patient care and support [11]. According to the research literature, the critical parameters for outcome measures for AD are clinical symptoms, the patient’s quality of life, and long-term follow-up [12]. Sometimes, it is difficult to determine how much disease severity affects AD patients and their quality of life; there are many instruments for assessing disease severity, but they are primarily used in clinical studies and rarely used in clinical practice. Among the most useful instruments, the Scoring Atopic Dermatitis (SCORAD) index and the Eczema Area and Severity Index (EASI) stand out. SCORAD is the most commonly used and best-validated AD severity assessment scale because it includes objective and subjective indicators of the disease [12]. Appropriate validity, response speed, reliability among operators, and interpretability, and the unclear reliability of intraobservers, have all been established for SCORAD [12]. Apart from the measurement of disease severity, there are also other factors possibly influenced by, and related to, AD patient quality of life, such as the current stage of disease (exacerbation or remission), disease perception, and patients’ personality features. All these factors and data on the burden of AD are important for clinicians and their assessment and management of AD in the clinical setting.

## 2. Materials and Methods

To obtain insight into the various factors associated with AD patient quality of life, we wanted to evaluate patient quality of life in relation to disease severity, patient disease perception, and personality traits. In addition, we wanted to compare data on AD patient quality of life between symptomatic patients (patients with current AD manifestations) and asymptomatic patients (in remission), as well as examine prominent features of quality of life after follow-up with the symptomatic AD patients. We hypothesized that AD patients’ quality of life is significantly related to their disease severity, disease stage (active (symptomatic) stage or remission), perception of the disease, and personality features.

The study was conducted at the Department of Dermatovenereology of the Sestre Milosrdnice University Hospital Center in Zagreb, Croatia with approval from the hospital’s Ethical Committee (number: EP-20098/16-4). Our subjects were AD patients who came to see a dermatologist at the above institution and who met the criteria for an AD diagnosis according to the Hanifin and Rajka criteria [13]. After an examination by a dermatologist, patients were asked if they would like to participate in the study and signed a written informed consent form.

For patients who had lesions/symptoms of AD (“symptomatic AD”), disease severity was assessed with the SCORAD index. All respondents then completed psychological questionnaires. Ultimately, 84 subjects participated in the study, including an equal number of patients with clinical manifestations of AD (42 patients) and those in remission (the control group) (42 patients). Patients in remission were considered asymptomatic if they had been free of AD symptoms for the month prior to their examination (visit) by a dermatologist.

In addition to an initial analysis of parameters for all patients (symptomatic and asymptomatic AD patients), we also conducted an additional prospective study of symptomatic AD patients at a two-month follow-up. Two months later, in symptomatic patients, all parameters were analyzed again except CCEI (which presents features of a person that do not quickly change).

### 2.1. Assessment of Disease Severity by SCORAD

A dermatologist assessed disease severity and SCORAD results. SCORAD considers the objective and subjective indicators of AD. The clinical features used to assess disease severity are erythema, swelling, oozing/crusting, scratch marks, skin thickening (lichenification), and dryness. Each characteristic is rated with 0, 1, 2, or 3, depending on severity. The extent of the disease is estimated according to the percentage of skin affected with eczema. SCORAD assesses subjective symptoms, i.e., the intensity of the itch and sleeplessness, on a scale from 0 (no symptoms) to 10 (maximum) and refers to the previous three days [14]. The numerical data on all three aspects of the disease are entered into tables and summed, with a maximum score of 103. A SCORAD result of less than 25 is marked as mild disease; 25 to 50 as moderate disease severity; and greater than 50 as severe AD. Asymptomatic patients have a SCORAD result of 0.

### 2.2. Questionnaires on Quality of Life (Psychological Tests)

#### 2.2.1. Dermatological Quality of Life Analyzed by the Dermatology Life Quality Index (DLQI)

The Dermatological Life Quality Index (DLQI) is often used to assess the condition-specific dermatological quality of life of dermatology patients. It consists of six subscales (10 facets in total) for assessing the limiting effects of dermatological disease on the daily lives of patients in individual domains during the previous week (symptoms and feelings, daily activities, leisure, work/school, personal relationships, treatment) [15]. Several answers are offered for each question (“very much”, “much”, “little”, “not at all”, and “does not apply to my case”). The total score is the sum of the scores for each question, where a higher score indicates a greater impact of dermatological disease on quality of life. The overall score ranges from 0 (absence of disease impact) to 30 (maximum impact), where 0–1 is considered no impact, 2–5 is a small impact, 6–10 is a moderate impact, 11–20 is a large impact, and 21–30 is an extremely large impact.

#### 2.2.2. Health-Related Quality of Life Analyzed by the World Health Organization Quality of Life Brief Version (WHOQLO-BREF)

For the assessment of general health-related quality of life, we used the short quality of life questionnaire, which assesses four main areas (domains): physical health, mental health, social relations, and the environment [16]. The World Health Organization Quality of Life Brief Version (WHOQOL-BREF) is a shorter version of the WHOQOL-100 questionnaire and is used when test time is limited. It contains 26 questions in total—24 questions from the original questionnaire (one from each of the 24 facets of quality of life) plus 2 questions from the Overall Quality of Life and General Health facet. Respondents are asked to look back over the previous two weeks and mark answers based on a Likert-type scale from 1 to 5, where 1 denotes the lowest agreement and 5 the highest agreement. Scores are calculated for each of the four domains and are expressed as the average response to the facets describing them. The question concerning a patient’s perception of their general quality of life and the question concerning the patient’s perception of their general health are considered separately. The answers are then transformed to a 0–100 scale. The reliability of the questionnaire is relatively high, which means that the domains of physical and mental health and the environment have a reliability coefficient > 0.75, while the domain of social relations is on the border with moderate reliability (0.68).

### 2.3. Illness Perception Analyzed by the Brief Illness Perception Questionnaire (Brief IPQ)

The Brief Illness Perception Questionnaire (Brief IPQ) contains nine parts and is designed to quickly evaluate patients’ cognitive and emotional perceptions of the disease. It was developed by forming one question that best summarizes each subscale of the BriefIPQ questionnaire that initially contained 80 facets. There are eight questions of a quantitative type: five on cognitive perception of the disease, one on understanding of the disease, and two on emotional components associated with the disease (fear, anger, depression). The five facets related to cognitive perception of the disease include consequences (expected effects and consequences of the disease), duration (how long the patient thinks the disease will last), personal control over the disease (to what extent the patient believes they can control the disease), therapy control (to what extent the patient believes they can recover), and identity (how a person identifies themselves concerning the disease and its symptoms). The test is short and understandable [17]. On a scale from 0 to 10, respondents indicate the extent to which they perceive each of the listed items. Each part represents an individual result. Answers are grouped into qualitative categories according to their content.

### 2.4. Personality Traits Analyzed by the Crown–Crisp Experiential Index (CCEI)

The Crown–Crisp index (CCEI) of experience identifies and measures common symptoms and personality traits within conventional categories of psychoneurotic diseases and personality disorders [18,19]. It is used in triage examinations, monitoring changes caused by specific therapeutic procedures, or for research purposes. According to its structure, the CCEI contains 48 facets arranged in six subscales: free-floating anxiety (feeling of indefinite fear, horror, excessive tension, and even panic) (FFA); phobic anxiety (the patient feels frightened only in certain situations, e.g., indoors, height, a crowd) (PHO); obsession (excessive meticulousness, punctuality, aversion to sudden changes) (OBS); somatic manifestations of anxiety (headache, shortness of breath, palpitations, insomnia, fatigue) (SOM); depression (gloom, sadness, difficulty thinking, slowness of action, lack of energy (DEP); and hysteria (“personal superficial and changeable sensibilities and overly dependent on others”) (HYS).

For each part, the respondent marks the answer that best describes them. The questions are easy to understand, making the index appropriate for respondents of different educational backgrounds and intellectual abilities. The overall score gives a measure of general emotional instability or neuroticism with a profile of six scores per subscale.

### 2.5. Statistical Methods

The Kolmogorov–Smirnov and Shapiro–Wilk tests were used to assess the data distribution. For variables that did not have a normal distribution, the median and interquartile range were used in the description of central tendency and variability measures. The Mann–Whitney U test was used in the analysis of quality of life dimensions, personality characteristics, and health perceptions between symptomatic and asymptomatic subjects. The effect size (as a measure of the size of differences between groups) was estimated using the formula r = Z/√N. For those with a normal distribution, the arithmetic mean and standard deviation and the t-test for independent samples were used, while effect size was calculated by the formula r = √ (t2/(t2 + df)). The t-test for dependent samples and the Wilcoxon test with calculation of the effect size by using the listed formulas were used for differences in SCORAD, psychometric characteristics, and cortisol levels between two periods. The following criteria were used to interpret the effect size: r < 0.1 = insignificant effect size; 0.1–0.3 = small effect size; 0.3–0.5 = moderate effect size; 0.5–0.7 = large effect size; and >0.7 = very large effect size [20]. Correlations (Spearman’s when the data did not have a normal distribution and Pearson’s when they did) and linear regression were performed to analyze the relationship between stress levels and the dimensions of psychometric instruments. For the interpretation of correlation strength, the criteria previously stated for the effect size were used. All analyses were performed with the commercial IBM SPSS 22 software (IBM Corp, Armonk, NY, USA).

## 3. Results

Of the total sample of 84 AD patients, 58 were women (69%) and 26 were men (31%). The asymptomatic AD group had more women than the symptomatic group (80% vs. 60%), but the difference was not statistically significant. Subjects’ ages ranged from 18 to 50 years (the interquartile age range was from 23 to 37 years), with an average age of 30 years for both the symptomatic and asymptomatic groups.

### 3.1. Results from SCORAD, DLQI, WHOQOL-BREF, Brief IPQ, and the CCEI Analyzed for Both Symptomatic and Asymptomatic AD Patients

SCORAD values for the symptomatic group ranged between 6.95 and 75.8, i.e., most patients had moderate AD; the mean SCORAD value was 33.66. A total of nine patients had a SCORAD result greater than 50 (severe AD). Patient age did not correlate with SCORAD values and quality of life (DLQI, WHOQOL-BREF).

**Figure 1:**
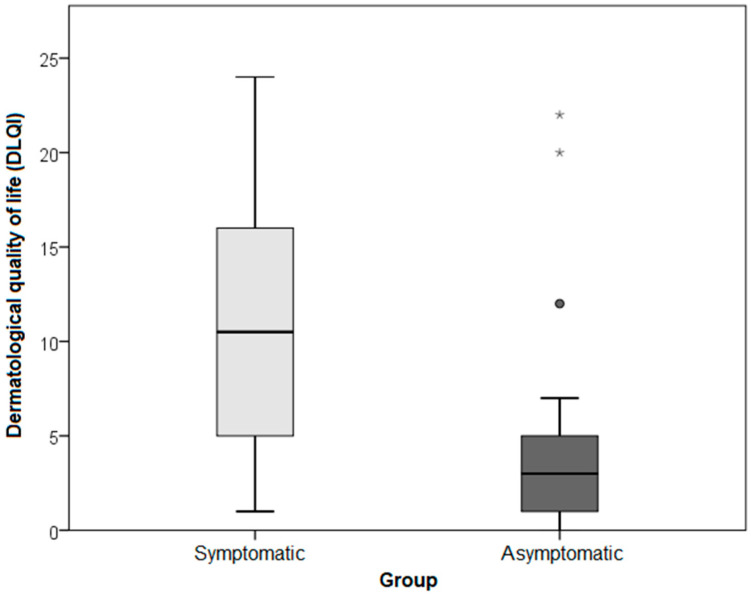
Dermatological quality of life impairment (DLQI) results for the symptomatic and asymptomatic AD groups. Circles and astersisk represent outleyers and extremes.

**Figure 2:**
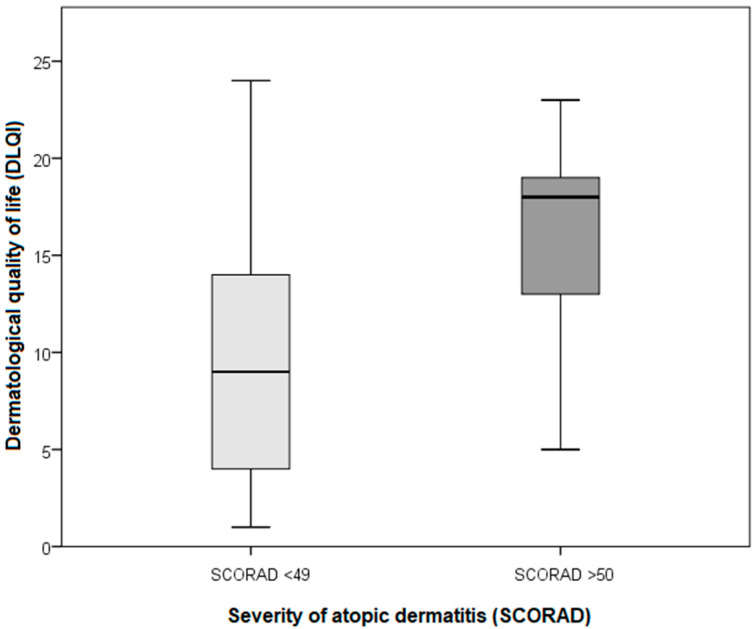
Comparison of the patient groups by disease severity (SCORAD) (mild and moderate AD (SCORAD < 49) and severe AD (SCORAD > 50)) and DLQI indices of the symptomatic AD patients.

Patient quality of life dimensions (DLQI, WHOQOL-BREF) are shown in Table 1, which shows data for the total sample, both symptomatic and asymptomatic AD patients together. Results specific to each group (symptomatic and asymptomatic) by disease severity (SCORAD) are presented in Table 2 and Figure 1 and Figure 2.

**Figure 3:**
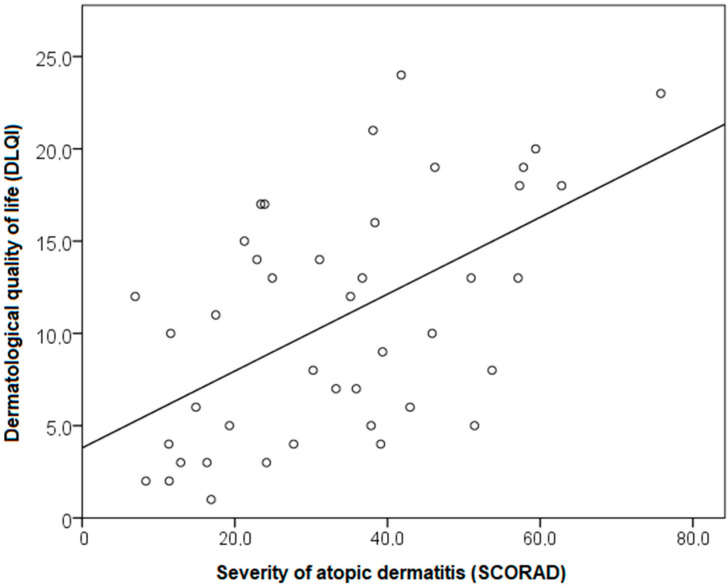
Relationship between the DLQI and the SCORAD index of the symptomatic AD patients.

In symptomatic AD patients, patients’ SCORAD values correlated with DLQI (r = 0.551; *p* < 0.001); this correlation was positive, linear, and large. The regression equation showed that an increase in SCORAD value by one scalar point resulted in a DLQI impairment increase by 0.2 scalar points (DLQI = 3.8 + 0.2 × SCORAD; R^2^ = 0.308; Adjusted R^2^ = 0.291; *p* < 0.001) (Figure 3). Eczema severity explains 30% of the variability in the DLQI.

The analysis of the WHOQOL-BREF questionnaire results for the symptomatic and asymptomatic groups (the data from Table 2) showed no significant differences between the two groups concerning the dimensions of the WHOQOL-BREF. Nevertheless, symptomatic AD patients had a significantly more impaired DLQI than asymptomatic patients, with a large effect size (*p* < 0.001; r = 0.565) (Table 2 and Figure 1).

A comparison of perception of illness and personality traits (CCEI) between symptomatic and asymptomatic AD patients were examined in a recent study by Meštrović-Štefekov et al. (in press) [21]. They found that symptomatic AD patients had significantly higher values for illness duration, symptoms, and worries than asymptomatic patients (Table 3). Additionally, a greater perception of disease was recorded for most of the IPQ dimensions for the symptomatic patients (previously reported, Meštrović-Štefekov et al., in press) [21]. Their disease severity (SCORAD) significantly correlated (positively and linearly) with the dimensions of impact of the disease on life, disease control, and symptoms (IPQ1, IPQ2, and IPQ5). In addition, when analyzing specific segments of the Brief IPQ, a significant decrease was seen in illness duration, symptoms, and understanding of the illness.

Symptomatic and asymptomatic AD patients did not, however, differ significantly in personality characteristics (measured by the CCEI).

**Figure 4:**
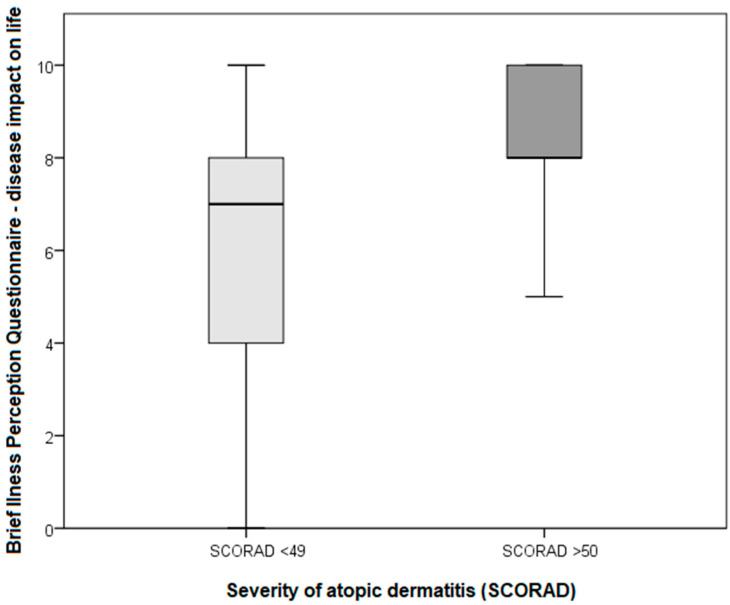
Comparison of SCORAD and IPQ1 indices of the symptomatic AD patients.

When looking at the relationship between IPQI, DLQI, and SCORAD, we found that patients with severe AD (SCORAD > 50), compared with those with mild and moderate AD (SCORAD < 49), had higher scores for disease impact on life (IPQ1) and the degree of quality of life impairment (DLQI) (Figure 2 and Figure 4).

In addition to SCORAD, DLQI was related to certain personality characteristics (free-floating anxiety disorder (FFA), obsession (OBS), somatization (SOM), and depression (DEP)), and Spearman’s correlations were positive, linear, and moderate (r = 0.332–0.437; *p* ≤ 0.032). Gender and age did not play a significant role. Multiple linear regression showed that when controlling for disease severity (SCORAD), additional significant predictors of DLQI were anxiety (FFA) and obsession (OBS) (Table 4). Other personality traits were not quality of life predictors. In describing the variability of DLQI values, the unique contribution of SCORAD was 42%, while those of anxiety and obsession were 6% and 5%, respectively. The whole model explains 56% of the variability. According to the results of the regression equation, with an increase in SCORAD values by one scalar point, quality of life impairment (DLQI) increased by 0.2; with an increase in anxiety by one point, DLQI increased by 0.7; and with increasing obsession, DLQI rose by 0.5 scalar points. DLQI impairment increased the most with increasing anxiety.

In symptomatic AD patients, SCORAD also correlated with disease impact on life, disease control, and disease symptoms, assessed by a short disease perception questionnaire (IPQ1, IPQ3, and IPQ5) (r = 0.350–0.398; *p* ≤ 0.023); this correlation was positive, linear, and weak. The SCORAD index was not related to personality traits.

### 3.2. Results for the Second Assessment of Parameters/Variables Analyzed at the Two-Month Follow-Up (Symptomatic Patients only) (SCORAD, DLQI, WHOQOL-BREF, and Brief IPQ)

The analysis of symptomatic AD patients after two months showed a significant decrease in patient SCORAD values and DLQI, with a moderate effect size (*p* ≤ 0.046; r = 0.306–0.398; Table 5). While there were no major changes in WHOQOL-BREF results, changes in Brief IPQ results were recorded (reported previously) [21]. Aside from a lower SCORAD and DLQI, a significant decrease in illness duration, symptoms, and understanding of illness was seen at the two-month follow-up, and an effect of illness on emotional state was recorded, with a small to moderate effect size (*p* ≤ 0.019; r = 0.255–0.340).

## 4. Discussion

Quality of life is a multidimensional term/concept that encompasses many facets of human wellbeing, including physical, environmental, and emotional health. The presence of disease can severely limit both specific and broad dimensions of daily functioning, significantly lowering a person’s quality of life. AD is known to be a significant burden on patients and their families and also for society [22]. According to the research literature, the adverse impact of AD on a patient’s quality of life and their psychological functioning can be compared with the impact of severe diseases, such as arthritis, diabetes, heart and malignant diseases, and depression [22,23,24,25,26]. Patients with AD are even more often absent from work than people with other chronic diseases, such as diabetes and hypertension. Thus, the quality of life of patients with moderate and severe AD, compared with other chronic diseases, is significantly lower and is usually associated with a lower overall health and life satisfaction score, impaired dermatological quality of life, and impaired quality of life associated with mental health [6]. Various features of AD contribute to the reduced quality of life [6,22,23,24,27]. Studies have shown that, just like other chronic, recurrent diseases, AD affects psychological, psychosocial, and professional aspects of life [22]. In addition to visible skin lesions, a significant burden is caused by the accompanying itching and need for scratching, where the scratching reflex itself further worsens the itching and dermatitis. Of all the clinical symptoms of AD, itching (pruritus) is most strongly associated with anxiety [28]. Consequently, a self-renewing cycle of itching and scratching occurs, which often puts the patient in a state of anxiety and reduces their quality of life [22,27,29,30]. In addition to skin and subjective symptoms, physiological disorders have also been demonstrated in AD patients who, compared with healthy subjects, have an elevated heart rate (at rest) that remains elevated in the case of itching, scratching, and mental stress [31,32]. Due to the chronic nature of AD, over time patients often face several disorders and difficulties, which can vary considerably depending on the type, severity, and patient’s perception of the disease. According to a recent study by Huang and analysis of data documenting quality of life impairment in AD patients and their families, aspects of quality of life impacted to a greater extent included symptoms of itching, feelings of embarrassment, and sleep disturbances [1]. In other studies, a higher degree of anxiety, anger, and concern has been observed in AD patients, which explains their lower quality of life compared with healthy subjects [23,25]. It has also been shown that patients with AD have an even higher degree of anxiety than patients with psoriasis [23]. In addition, studies on adult patients show an association between AD, depression, and anxiety, and a positive correlation between AD and depression has even been recorded in children [33].

Research has been conducted in various countries on AD’s impact on quality of life, specifically identifying factors that affect the degree of quality of life impairment. In determining the impact of AD on quality of life, psychological functioning, and overall health, many psychometric tests are used to assess and measure the patient’s perception of their physical, emotional, social, environmental, and other segments of life and factors that may affect, or result from, the disease. In our study, we used the DLQI, the most widely used tool to assess the patient’s perspective of their disease severity. We also wanted objective data and thus used the SCORAD as well, the best-validated tool for AD severity assessment and a widely used tool for clinician-reported disease severity [34,35]. According to recent results by Huang, et al., AD severity affects the degree of impairment of quality of life, but with no apparent link between quality of life impairment and patient demographic or medical factors (age at diagnosis or duration of illness) [1]. Our study showed similar results, where quality of life (DLQI) was dependent on disease severity (SCORAD), exhibiting a significant, highly positive correlation, and was influenced by patient personality characteristics (free-floating anxiety disorder, obsession, somatization, and depression). Significant predictors of quality of life (DLQI) were disease control perception (IPQ3), anxiety, and obsession; other personality traits were not quality of life predictors. In addition, dimensions of patient quality of life estimated by the WHOQOL-BREF had moderate to good reproducibility. In clinical practice, AD patient personality features need to be taken into account, as they may be related to their quality of life and the specific approach to them. In addition, the intensity of patient depression and perception of disease control (IPQ3) increased with age. Thus, when working with AD patients of older age, it is important to be aware that they are more prone to these concomitant issues and may need psychological support to ameliorate their AD condition and improve their quality of life. However, the limitation of our study is the small number of enrolled examinees—we hope that studies with a higher number of AD patients would be performed in the future. Additionally, specific personality characteristics (e.g., in AD patients) are hard/difficult to change (interventions focusing on altering personality traits are almost impossible, as they tend to be stable over time) but they should be recognized and should indicate to clinicans the specific approach to be taken, including psychological support when necessary. Further research in this specific area could provide useful data.

The correlation between severity of clinical AD features and reduced quality of life has been analyzed by a handful of studies [22,27,36]. According to most of them, the impact of AD on patients’ quality of life is unexpectedly strong. They note that a patient’s perception of the disease is significant because it encompasses various experiences (e.g., sadness, coping with their illness, functional impairment), something that is true for other chronic diseases and conditions as well. However, with many chronic conditions, this does not necessarily hold true and there appears to be no correlation between a person’s perception of their condition and the actual severity of the condition—the patient’s perception is strongly influenced by their experience of and the meaning they give to the disease rather than the actual severity of the disease [27]. Perception and experience of the disease also depend on the patient’s psychological characteristics and personality. Often, the importance that the affected person attaches to their illness is more important than the objective severity of the illness [27]. Therefore, clinicians and researchers need to take into account the fact that perception of illness and quality of life assessments depend on various factors, such as personal and family history, environment, family and friends, social support, and other areas of life that are not directly related to AD [36]. Furthermore, psychological disturbances as a consequence of AD are common in AD patients, primarily anxiety and depression [37,38]. According to recent data, a high prevalence (40%) of anxiety and depression is observed in AD patients compared with the general population (17.5%) [37]. According to a study by Silveberg et al., symptoms of anxiety and depression have been observed in almost all patients with moderate to severe AD, which should be considered when assessing a patient’s condition [37]. Apart from an increased risk of developing depression and anxiety, even suicidal thoughts have been recorded among adult AD patients, as shown by one cohort study [38]. According to other study results, AD patients have a significantly increased risk of suicidal thoughts and suicide attempts and a higher risk of having/developing various mental health disorders, such as ADHD, as well as behavioral disorders and autism [8,9,10,39].

Given these findings, the medical community has looked at how to mitigate the influence of AD features and concomitant factors and psychologicl disturbances (e.g., itching, anxiety, and depression) to properly treat the underlying disease. Useful treatment methods include integrative body and mind training (IBMT), which uses aspects of awareness and meditation to effect physiological changes, including oxygen consumption, heart rate, skin conduction, and respiratory rate (during IBMT, the heart rate slows down, heart rate variability is high, and the respiratory system amplitude increases) [32]. Other methods such as autogenic training, cognitive-behavioral therapy, and stress management methods may also be helpful to patients [31]. For severe forms of AD accompanied by intense itching, tricyclic antidepressants have been used for decades. Studies have shown that medicines that alleviate psychological stress, such as selective reuptake inhibitors, olopatadine (a selective histamine H1 receptor antagonist), aprepitant (a selective neurokinin receptor one (NKR1) antagonist), and bupropion (an antidepressant that inhibits the reuptake of norepinephrine and dopamine), have also shown promising results in combating pruritus and improving AD; however, additional studies are needed to confirm their efficacy and safety [31,40,41,42,43]. In addition, support from family, friends, and colleagues and having a positive attitude are also important factors influencing patient quality of life.

To the best of our knowledge, this is the first study to compare the quality of life and related features of patients in the active stage of AD to those in remission. Symptomatic AD patients had a significantly more impaired DLQI than asymptomatic patients, and the groups differed in some IPQ dimensions, but they did not significantly differ concerning the dimensions of the WHOQOL-BREF and personality traits (CCEI). For symptomatic patients, DLQI correlated with AD severity (SCORAD) and was related to certain personality characteristics (free-floating anxiety disorder, obsession, somatization, and depression). Our results indicate that, while AD severity positively correlated with some dimensions of impact of AD on quality of life, patient perception of control over the disease, and symptoms, other parameters showed very similar results for both the patients in the active stage of disease and those in remission. Together, these results suggest that general patient quality of life is affected by AD but that the effects are only somewhat different between those with manifestations and those without, namely that those in the active stage of disease were affected by their skin manifestations. Effects on patient personality characteristics were also largely similar, most likely because those in remission still deal with the burden and the risk that manifestations will reappear.

## 5. Conclusions

The chronic, recurrent, and debilitating nature of AD significantly reduces patient quality of life, often causing itching, sleep disorders, and consequent anxiety and depression. It is challenging but necessary to recognize and understand the various factors affecting the type and severity of each patient’s AD, also taking into account that there are also many indirect disease-related issues to address. Our results indicate that AD patient quality of life is dependent not only on disease severity but is also influenced by patient personality characteristics and their concomitant psychological disturbances (anxiety disorder, obsession, somatization, depression). In other words, AD patient quality of life is related to many different factors and is partially influenced by disease stage since even patients without current clinical AD manifestations (in remission) are always at risk of a recurrence, which itself can negatively impact their quality of life and cause concomitant psychological disturbances. Thus, it is necessary to take them into account when analyzing AD patients’ quality of life predictors. All these findings suggest that improving AD patients’ quality of life requires clinicians to consider a multidisciplinary treatment approach with psychological support strategies to create effective, patient-specific therapy regimens.

## Figures and Tables

**Table 1:** Patients’ quality of life characteristics (DLQI, WHOQOL-BREF) analyzed for both symptomatic and asymptomatic AD patients together (total sample, N = 84).

	N	Mean	SD	Median	IQR	Min	Max
Dermatology Life Quality Index (DLQI)	84	7.4	6.5	5	2.25–12	0	24
Health-Related Quality of Life (WHOQOL-BREF)							
Physical health	84	72.62	14.29	71.43	64.23–82.14	39.29	100
Mental health	84	69.68	16.28	70.83	59.38–83.33	33.33	95.83
Social relations	84	76.54	17.22	75	66.67–91.67	33.33	100
Environment	84	72.54	13.94	71.88	62.5–84.38	40.63	100

N, sample size; SD, standard deviation; IQR, interquartile range; Min, minimal value; Max, maximal value.

**Table 2:** Quality of life dimensions (DLQI, WHOQOL-BREF) of symptomatic and asymptomatic AD patients (total sample, N = 84).

	Group	Average	SD	Median	IQR	*p*	r
Dermatology Life Quality Index	symptomatic	10.8	6.4	10.5	5–16.25		
(DLQI)	asymptomatic	4.0	4.6	3.0	1–5	<0.001	0.565
Physical health	symptomatic	72.45	14.47	75.0	66.1–82.1		
(WHOQOL)	asymptomatic	72.79	14.28	71.4	63.4–82.1	0.907	0.013
Mental health	symptomatic	70.63	16.20	75.0	61.5–80.2		
(WHOQOL)	asymptomatic	68.73	16.49	70.8	58.3–83.3	0.575	0.061
Social relations	symptomatic	79.46	17.47	83.3	66.7–93.8		
(WHOQOL)	asymptomatic	73.61	16.66	75.0	58.3–85.4	0.108	0.176
Environment	symptomatic	73.68	13.51	75.0	65.6–84.4		
(WHOQOL)	asymptomatic	71.40	14.43	68.8	61.7–85.2	0.473	0.078

**Table 3:** Patient perception of disease results for symptomatic and asymptomatic AD patients (total sample, N = 84).

	Group	Average	SD	Median	IQR	*p*	r
Illness impact on life	symptomatic	6.4	2.7	7.0	5–8		
(IPQ1)	asymptomatic	4.7	2.8	4.5	3–7	0.004	0.316
Illness duration	symptomatic	8.6	1.8	9.0	7.75–10		
(IPQ2)	asymptomatic	5.8	3.1	6.0	4–8	<0.001	0.460
Illness control	symptomatic	4.6	2.8	5.0	2–7		
(IPQ3)	asymptomatic	5.1	2.4	5.0	3–7	0.376	0.097
Illness treatment	symptomatic	7.2	2.2	7.0	5–9		
(IPQ4)	asymptomatic	7.7	2.3	8.0	7–9	0.150	0.157
Illness symptoms	symptomatic	7.9	2.2	8.0	7–10		
(IPQ5)	asymptomatic	5.2	2.5	5.0	3–7	<0.001	0.521
Worries about the illness	symptomatic	7.5	2.8	8.0	6–10		
(IPQ6)	asymptomatic	5.1	2.9	5.0	3–7	<0.001	0.403
Understanding of the illness	symptomatic	7.2	2.7	8.0	5–10		
(IPQ7)	asymptomatic	6.0	2.9	6.0	4–8	0.041	0.223
Effect of the illness on emotional state	symptomatic	6.6	2.8	7.0	5–9		
(IPQ8)	asymptomatic	5.0	2.9	5.5	2–7	0.009	0.287

**Table 4:** SCORAD values and personality traits as quality of life (DLQI) determinants for the symptomatic AD patients (N = 42).

	Non-Standardized Coefficient		Standardized Coefficient	*p*	Correlations		
	B	SE	Beta		Zero Order	Partial	Semipartial
Constant	−4.147	2.2					
SCORAD	0.2	0.0	0.7	<0.001	0.555	0.721	0.646
FFA	0.7	0.3	0.4	0.024	0.411	0.365	0.243
OBS	0.5	0.3	0.3	0.046	0.351	0.326	0.214
SOM	0.1	0.3	0.0	0.891	0.310	0.023	0.014
DEP	−0.1	0.4	−0.0	0.855	0.366	−0.031	−0.019

R = 0.785; R^2^ = 0.615; Adjusted R^2^ = 0.562; *p* < 0.001.

**Table 5:** Comparison of results of the first (1) analysis and the second (2) analysis (at the 2-month follow-up) for symptomatic patients: DLQI; SCORAD; physical health; mental health; social relations; and environment (DLQI, WHOQOL-BREF).

	Mean	SD	Median	IQR	*p*	r
DLQI 1	10.8	6.4	10.5	5–16.3		
DLQI 2	9.2	6.6	9	3–14.3	0.046 *	0.306
SCORAD 1	33.66	17.07	34.20	18.85–45.94		
SCORAD 2	20.20	22.39	16.78	0.00–37.59	<0.001	0.398
Physical health 1	72.45	14.47	75.00	66.07–82.14		
Physical health 2	73.27	15.07	76.79	60.71–83.04	0.536	0.068
Mental health 1	70.64	16.20	75.00	61.46–80.21		
Mental health 2	72.32	16.01	70.83	61.46–84.38	0.217	0.135
Social relations 1	79.46	17.47	83.33	66.67–93.75		
Social relations 2	82.14	15.79	83.33	75.00–93.75	0.081	0.190
Environment 1	73.68	13.51	75.00	65.63–84.38		
Environment 2	74.03	11.80	71.88	65.63–81.25	0.802 *	0.039

* *t*-test for dependent samples.
